# Antiphospholipid antibodies in patients with stroke during COVID-19: A role in the signaling pathway leading to platelet activation

**DOI:** 10.3389/fimmu.2023.1129201

**Published:** 2023-03-02

**Authors:** Antonella Capozzi, Gloria Riitano, Serena Recalchi, Valeria Manganelli, Agostina Longo, Anne Falcou, Manuela De Michele, Tina Garofalo, Fabio M. Pulcinelli, Maurizio Sorice, Roberta Misasi

**Affiliations:** ^1^ Department of Experimental Medicine, “Sapienza” University of Rome, Rome, Italy; ^2^ Emergency Department, “Sapienza” University of Rome, Rome, Italy

**Keywords:** antiphospholipid antibodies, β2-GPI, COVID-19, thrombosis, stroke.

## Abstract

**Background:**

Several viral and bacterial infections, including COVID-19, may lead to both thrombotic and hemorrhagic complications. Previously, it has been demonstrated an “*in vitro*” pathogenic effect of “antiphospholipid” antibodies (aPLs), which are able to activate a proinflammatory and procoagulant phenotype in monocytes, endothelial cells and platelets. This study analyzed the occurrence of aPL IgG in patients with acute ischemic stroke (AIS) during COVID-19, evaluating the effect of Ig fractions from these patients on signaling and functional activation of platelets.

**Materials and methods:**

Sera from 10 patients with AIS during COVID-19, 10 non-COVID-19 stroke patients, 20 COVID-19 and 30 healthy donors (HD) were analyzed for anti-cardiolipin, anti-β2-GPI, anti-phosphatidylserine/prothrombin and anti-vimentin/CL antibodies by ELISA. Platelets from healthy donors were incubated with Ig fractions from these patients or with polyclonal anti-β2-GPI IgG and analyzed for phospho-ERK and phospho-p38 by western blot. Platelet secretion by ATP release dosage was also evaluated.

**Results:**

We demonstrated the presence of aPLs IgG in sera of patients with AIS during COVID-19. Treatment with the Ig fractions from these patients or with polyclonal anti-β2-GPI IgG induced a significant increase of phospho-ERK and phospho-p38 expression. In the same vein, platelet activation was supported by the increase of adenyl nucleotides release induced by Ig fractions.

**Conclusions:**

This study demonstrates the presence of aPLs in a subgroup of COVID-19 patients who presented AIS, suggesting a role in the mechanisms contributing to hypercoagulable state in these patients. Detecting these antibodies as a serological marker to check and monitor COVID-19 may contribute to improve the risk stratification of thromboembolic manifestations in these patients.

## Introduction

Several infections have been shown to play a role in the triggering of anti-phospholipid antibodies (aPLs) (1, 2); indeed, it was demonstrated an overall increased risk of developing anti-cardiolipin (aCL) antibodies and an association between viral infections and presence of anti-β2-Glycoprotein I (anti-β2-GPI) antibodies (3). aPLs are often transient during infections, but in some cases, they may be associated with thromboembolic events (4). The most common mechanism involved in the infectious origin of the aPLs is molecular mimicry between β2-GPI and/or other Antiphospholipid Syndrome (APS) antigens and infectious agents (5). Another well-studied mechanism is the two-hit hypothesis, which attempts to explain the persistent presence of aPLs without the occurrence of thrombotic events. Detection of aPLs might represent the first hit, whereas several findings underline the role of infections as a potential second hit. This hypothesis is supported by data that demonstrate, at the same time, the involvement of innate immunity receptors in APS pathogenesis mechanism(s) (6). The association between aPLs and infectious agents was first described in syphilis, but many other viral, bacterial and parasitic infections have been found to induce aPLs. The most common ‘‘triggering’’ factors are skin infections, human immunodeficiency virus (HIV), pneumonia, hepatitis C virus (HCV), hepatitis B virus (HBV) (7–9).

In patients affected from coronavirus disease 2019 (COVID-19) thromboembolic events involving arterial, venous and microcirculation have been frequently reported. Therefore, this syndrome is now described as a viral pulmonary infection with respiratory complications, and more precisely as a multiple-organ disorder accompanied by hypercoagulability (10–12).

Furthermore, it has been reported that, even in young patients, neurological manifestations, in particular ischemic stroke, may arise in the context of COVID-19 (13–15). Interestingly, some papers have described the presence of aPLs together with increasing proinflammatory cytokine levels in COVID-19 patients with ischemic stroke (16). aPLs, including IgG and/or IgM aCL antibodies, IgG and/or IgM anti-β2-GPI antibodies, and Lupus Anticoagulant (LA) are established as laboratory criteria for diagnosis of the APS, characterized by arterial and venous thrombosis and recurrent abortions (17). In addition, some “non-criteria” aPLs have shown promising clinical utility, especially in “seronegative” APS (SN-APS) patients, with a clinical picture typical of APS, but persistently negative for routine aPL antibody tests. They include mainly anti-phosphatidylserine/prothrombin (aPS/PT) and anti-vimentin/CL (aVim/CL) antibodies (18).

Reports in COVID-19 patients described the association of aPLs with thrombotic events (19), demonstrating that injection of the serum IgG fraction from these patients into mice resulted in significant increase of thrombus formation in addition to neutrophil hyperactivity, higher platelet counts and lower clinical estimated glomerular filtration rate. The mechanisms underlying the prothrombotic state are more complex and still unclear, but likely related to platelets, acting as mediators of thrombosis and hemostasis, but also as immune mediator. In fact, in critically ill COVID-19 patients, platelets are hyperactivated with an excessive secretion of procoagulant molecules (20–22). Activated platelets also secrete platelet factor 4 (PF4), a member of the C-X-C chemokine family with high affinity for heparin and other anionic glycosaminoglycans (e.g., endothelial cell surface or platelet surface GAGs). PF4 has a proven procoagulant role but appears to have also anticoagulant effects (23, 24).

A role of platelets has been also demonstrated in aPL-related thrombosis by experimental models, which revealed that platelets are activated following the infusion of anti-β2-GPI antibodies (25). Platelet activation is increased when anti-β2-GPI–β2-GPI complexes bind to the platelet thrombus and the activation-signaling pathways are mediated by interaction with phospholipids on the cell surface (phosphatidylserine, phosphatidylethanolamine) or with receptors of platelet membrane (26, 27). Their combination in complexes contributes to the activation of two platelet receptors for β2-GPI, apolipoprotein E receptor 2’ (apoER2’) - and glycoprotein Ib α (GPIb α), that in turn cross-link anti-β2-GPI antibodies (28, 29). In our previous paper we showed that aPLs, in particular anti-β2-GPI antibodies, were able to induce intracellular signals in platelets, which involve IRAK phosphorylation and NF-κB activation, leading to the up-regulation of Tissue Factor (TF), the major initiator of the clotting cascade (30).

Moreover, anti-β2-GPI antibody binding induces the activation of a platelet prothrombotic phenotype expressed by p38 mitogen-activated protein kinases (MAPK) phosphorylation, GP IIb/IIIa conformational change, P-selectin expression, and thromboxane B2 production (31). Therefore, MAPK pathways, including ERK kinases, activated by various stimuli, are important intracellular signaling in the activation of platelets as pivotal component of arterial and venous thrombosis (31, 32).

Since COVID-19 infection triggers the production of aPLs and the thrombotic events observed in severe COVID-19 resemble hypercoagulation seen in APS, especially as regards the catastrophic variant, it is reasonable to assume that aPLs may be potential mediators of cerebrovascular events in patients with COVID-19. From this assumption, our study analyzed the occurrence of aPLs in patients with COVID-19 and concomitant acute ischemic stroke (AIS), evaluating the effect of Ig fractions from these patients on signaling and functional activation of platelets.

## Materials and methods

### Patients

We enrolled 10 patients, referred to Emergency Department of Umberto I Polyclinic of Rome, positive for PCR Sars-CoV-2 and affected by large vessel occlusion AIS [mean age 72.8 (S.D. 17.6); 7 males, 3 females]. Clinical and demographic characteristics of patients are reported in **Table 1**. As control groups, matched for age and sex, we evaluated 10 non-COVID-19 stroke patients [mean age 71.5 (S.D. 17.5); 6 males, 4 females], 20 COVID-19 patients without vascular thrombosis [mean age 68.5 (S.D. 15.8); 13 males, 7 females] and 30 healthy donors (HD) [mean age 65.0 (S.D. 16.2); 21 males, 9 females]. Sera were collected and stored at −20°C until use. This study was conducted in compliance with the Helsinki declaration and approved by the local ethic committees (number NCT04844632); participants gave written informed consent.

**Table 1:** Demographics and clinical characteristics of the COVID-19/AIS patients.

	AIS patients *n* =10
Demographics, vascular risk factors and pre-stroke medications	
Age, mean (SD)	72.8 (17.6)
Female/Male	3/7
Stroke clinical and radiological characteristics	
Stroke on awakening/unknown time of onset	2 (20)
Infarct location - Right Left - Bilateral - Subtentorial	4 (40) 6 (60) 0 0
Occlusion site - Top of ICA - Tandem occlusion - MCA-M1 - MCA-M2 - MCA-M3-M4 - Posterior circulation - No occlusion	1 (10) 0 4 (40) 3 (30) 0 0 2 (20)
NIHSS at baseline, median (IQR)	17 (4.75-23)
NIHSS at 24 h, median (IQR)	8 (4-19)
IV thrombolysis	1 (10)
Mechanical thrombectomy	4 (40)
Hemorrhagic transformation	3 (30)
TICI - 2a - 2b - 3	1/4 (25) 0 3/4 (75)
Infarct volume, cm^3^ - mean (SD) - median (IQR)	73.47 (108.25) 25.95 (7.80-105.35)
Characteristics of COVID-19	
Time from COVID-19 diagnosis and stroke (days) - mean (SD) - median (IQR)	6.5 (8.7) 4 (0-12.25)
Chest CT	9 (90)
Pneumonia - <20% (mild) - 20-50% (moderate) - >50% (severe) - Bilateral pleural effusion	9 (90) 1/9 (11.1) 3/9 (33.3) 3/9 (33.3) 2/9 (22.2)
SOFA	
- 0	1/9 (11.1)
- 1	2/9 (22.2)
- 2	2/9 (22.2)
- 3	2/9 (22.2)
- 4	2/9 (22.2)
Laboratory data (reference values)	
Lymphocytes, x10^3^/μL (1-3.2)	1.31 (0.81)
PLT, x10^3^/μL (150-450)	284.7 (112.41)
LDH, UI/L (135-225)	447.89 (340.54)
Myoglobin, ng/ml (28-72)	294 (283.95)
CRP, mg/dL (0-0.5)	6.59 (15.74)
D-dimer, μg/L (0-550)	3115.40 (1719.73)
Fibrinogen, mg/dL (200-400)	481.0 (91.35)
INR (0.8-1.2)	1.07 (0.09)
aPTT (0.8-1.2)	0.89 (0.18)

Values are expressed as means (SD) and percentages.

Ig fractions were isolated from sera of patients or healthy donors using (NH4)_2_SO_4_ (ammonium sulfate, Sigma-Aldrich, St Louis, MO, USA) precipitation (33), slightly modified. In particular, following a preliminary purity reduction step by precipitation with caprylic acid (octanoic acid, Sigma-Aldrich) (34), saturated (NH4)_2_SO_4_ to a final concentration of 33% was slowly added to sera and incubated for 1 h at 4°C. Then, the samples were centrifuged at 3000 x g for 30 min at 4°C and carefully the supernatant was decanted into a fresh tube. Again, saturated (NH4)_2_SO_4_ (one-third of the supernatant volume) was added to bring its final concentration to ∼50% and incubated for 1 h at 4°C. The resulting precipitated Ig were isolated by centrifugation at 3000 x g for 30 min at 4°C after removing the supernatant. The pellets were resuspended with PBS equal to the original volume of sera. Finally, samples were dialyzed overnight against (NH4)_2_CO_3_ (ammonium carbonate) through a dialyzing membrane to remove remaining ammonium sulfate, lyophilized and resuspended in sterile PBS.

### Detection of aPL antibodies

All the patients and HD sera were analysed for the presence of aCL and anti-β2-GPI antibodies (IgG and IgM) by ELISA using QUANTA Lite™ detection kit (INOVA Diagnostic Inc., San Diego, CA, USA) and confirmed by chemiluminescence assay using Zenit RA Immunoanalyzer (A. Menarini Diagnostics, Florence, Italy), according to manufacturer’s instructions.

### Detection of “non-criteria” aPL antibodies

All the sera were also tested for the presence of “unconventional” (“non-criteria”) aPLs: IgG and IgM antibodies specific for PS/PT were assessed by ELISA using a QUANTA Lite™ detection kit (INOVA Diagnostic Inc.) according to manufacturer’s instructions; IgG and IgM aVim/CL antibodies were tested by ELISA assay, as previously reported (18).

### Detection of anti-PF4 antibodies

We additionally tested patients and HD sera for antibodies against PF4/polyanion, using a commercial enzyme immunoassay (Immucor, Lifecodes, Waukesha, WI). Sera with an optical density > 500 arbitrary units (AU) were considered as positive.

### Platelet preparation

Platelets were obtained from blood samples [in presence of acid citrate dextrose (ACD) as anticoagulant] of healthy donors, that signed informed consent from Transfusional Center of Policlinico Umberto I, Sapienza University of Rome.

Platelet-rich plasma (PRP) was preliminary separated from the whole blood by centrifugation at 150 x g for 15 min at 20°C. Two thirds of the PRP, with the addition of ACD, to prevent platelet activation, were transferred into a new another sterile tube, without disturbing the buffy coat layer, in order to avoid contamination. PRP was centrifuged at 900 x g for 10 min at 20°C (with no brake applied). Platelet-poor plasma (PPP) was discarded and platelet pellets were resuspended in Tyrode’s buffer, containing 10% (v:v) ACD. Then, after washing, as above, platelets pellets were resuspended in Tyrode’s buffer, containing Bovine Serum Albumin (BSA, 3 mg/ml).

Platelet were counted by a hemocytometer (Coulter, Beckman Coulter, Brea, California, USA), which gives that leukocyte contamination was < 1 leukocyte/10^7^ platelets. The purity of the isolated platelets was analyzed and confirmed by staining with a fluorescein isothiocyanate (FITC)–conjugated anti-CD41 antibody (Beckman Coulter) and using flow cytometry (Coulter Epics, Beckman Coulter; data not shown).

### *In vitro* incubation of human platelets and western blot analysis

After isolation, human platelets (3 × 10^8^/ml), were seeded in into 6-well cell culture plates and incubated with Ig fractions (200 μg/ml) from sera of patients described above, with Ig fractions from sera of healthy donors, with polyclonal anti-β2-GPI IgG (200 μg/ml, Affinity Biologicals, Ancaster, Ontario, Canada) or with control IgG (Sigma Aldrich, cod. NI02, 200 μg/ml), for 10 min at 37°C, according to the methods previously described (35, 36). After the treatment, platelets were lysed in a buffer prepared with 20 mM HEPES, pH 7.2, 1% Nonidet P-40, 10% glycerol, 50 mM NaF, 1 mM Na_3_VO_4_ and protease inhibitors cocktail (Sigma-Aldrich). Protein extracts, in equal amount, were analyzed by western blot and, for this purpose, they were first subjected to 10% sodium dodecylsulfate polyacrylamide gel electrophoresis (SDS-PAGE) and then transferred onto polyvinylidene difluoride (PVDF) membranes (Bio-Rad Laboratories, Richmond, CA, USA). Membranes, after blocking with Tris-buffered saline Tween 20 (TBS-T) 3% BSA, were incubated with polyclonal rabbit anti-phospho-ERK1/2 (Cell Signaling, Inc., Danvers, MA, USA) and polyclonal rabbit anti-phospho-p38 antibodies (Cell Signaling, Inc.). Horseradish peroxidase (HRP)-conjugated anti-rabbit IgG (Sigma-Aldrich) and then enhanced chemiluminescence western blot system (Amersham Pharmacia Biotech, Buckinghamshire, UK) were used to visualize antibody reactions. As a control of loaded protein content, phospho-ERK1/2 and phospho-p38 membranes were stripped and reprobed with rabbit anti-ERK1/2 and rabbit anti-p38 (Cell Signaling, Inc.) respectively. Densitometric scanning analysis was performed using a NIH Image 1.62 software (National Institutes of Health). The density of each band (absolute value) in the same gel was analyzed.

### Platelet activation

Platelets from healthy donors were prepared as reported above. Platelets were incubated for 1 h, with a mix of agonists composed by U-446619 (1 μM; synthetic thromboxane A2 receptor agonist, Helena Biosciences Europe) plus epinephrine (10 μM; Helena Biosciences Europe) used as positive control, or, alternatively, with Ig fractions (200 μg/ml) from sera of patients or healthy donors and then analyzed by a luciferin/luciferase method (ATP lite, PerkinElmer, Waltham, MA) to analyze platelet secretion by ATP release dosage. ATP release was calculated as a percentage of max response from the U-446619 plus epinephrine positive control. This method was performed as previously reported (36–38).

In parallel experiments, platelet function was also investigated evaluating platelet aggregation. Briefly, after treatment, platelets were added in appropriate wells and, immediately after, the plate was placed in a Plate Reader Victor 3 (PerkinElmer). The absorbance and platelet aggregation (PA) percentage were assessed using the following formula: PA% = (sample Absorbance Units − PRP Absorbance Units)/(PPP Absorbance Units− PRP Absorbance Units) × 100, as previously described (36).

### Statistical analysis

For western blot analysis the statistical procedures were performed by GraphPad Prism software Inc. (San Diego, CA, USA). D’Agostino-Pearson omnibus normality test was used to assess the normal distribution of the data. Normally distributed variables were summarized using the mean ± standard deviation (SD). Differences between numerical variables were tested using Paired t-test.

For ATP dosage, the level of significance was determined by unpaired, 2-tailed Student’s t test by KaleidaGraph Software 3.6. Results (showed as mean + SD) are considered statistically significant: *p ≤ 0.05, **p ≤ 0.01 ***p ≤ 0.001, ****p ≤0.0001.

## Results

### Serum detection of antiphospholipid antibodies

The study included 10 patients defined as COVID-19/AIS. Clinical and demographic characteristics of patients are reported in **Table 1**.

This study analyzed the occurrence of aPLs in these patients. In particular, aCL IgG were detected in 5/10 (50%) patients and aCL IgM in 2/10 (20%). The patients who tested positive for IgG and IgM anti-β2-GPI antibodies were 3/10 (30%) and 2/10 (20%) respectively. All positive samples were confirmed after 12 weeks. Two out of 10 (20%) resulted positive for IgG aPS/PT antibodies; the prevalence of aVim/CL antibodies was of 5/10 (50%) for IgG (**Tables 2**, **3**).

**Table 2:** Positivity of aPL antibodies in the 10 patients with acute ischemic stroke during COVID-19.

Patient *n*	aCL IgG IgM (GPL) (MPL)		aβ2-GPI IgG IgM (UA/ml) (UA/ml)		aPS/PT IgG IgM (UA/ml) (UA/ml)		aVim/CL IgG IgM		ATP release (%)	Platelet aggregation (%)
1	47.0	neg	neg	neg	neg	neg	pos	neg	8	22
2	neg	neg	neg	neg	neg	neg	pos	neg	6	21
3	69.7	neg	32.1	neg	35.9	neg	pos	neg	10	20
4	59.6	55.6	28.6	25.0	neg	neg	pos	neg	11	32
5	141.6	neg	27.1	neg	38.0	neg	pos	neg	7	24
6	neg	neg	neg	neg	neg	neg	neg	neg	3	15
7	neg	neg	neg	neg	neg	neg	neg	neg	17	25
8	neg	neg	neg	neg	neg	neg	neg	neg	3	15
9	neg	neg	neg	neg	neg	neg	neg	neg	2	17
10	70.0	62.3	neg	32.1	neg	neg	neg	neg	16	29

Ig, immunoglobulin; aCL, anti-cardiolipin; aβ2-GPI, anti-β2-Glycoprotein I; aPS/PT, anti-phosphatidylserine/prothrombin; aVim/CL, anti-vimentin/cardiolipin.

The cut-off level used is 20 GPL or MPL units for aCL IgG or IgM, 15 UA/ml for aβ2-GPI IgG or IgM and 30 UA/ml for aPS/PT according to manufacturer’s instructions.

**Table 3:** Prevalence of autoantibodies in patients and healthy donors.

Autoantibodies	COVID-19/AIS (10) *n* (%)	Non-COVID-19/stroke (10) *n* (%)	COVID-19 (20) *n* (%)	Healthy donors (30) *n* (%)
**aCL IgG**	5 (50)	0 (0)	0 (0)	0 (0)
**aCL IgM**	2 (20)	0 (0)	2 (10)	0 (0)
**aβ2-GPI IgG**	3 (30)	0 (0)	0 (0)	0 (0)
**aβ2-GPI IgM**	2 (20)	0 (0)	2 (10)	0 (0)
**aPS/PT**	2 (20)	0 (0)	0 (0)	0 (0)
**aVim/CL**	5 (50)	0 (0)	0 (0)	0 (0)
**aPF4**	0 (0)	0 (0)	0 (0)	0 (0)

AIS, acute ischemic stroke; Ig, immunoglobulin; aCL, anti-cardiolipin; aβ2-GPI, anti-β2-Glycoprotein I;

aPS/PT, anti-phosphatidylserine/prothrombin; aVim/CL, anti-vimentin/cardiolipin; aPF4, anti-platelet factor 4.

In the control group of non-COVID-19 stroke no patients were positive for the aPL tests used, whereas in the COVID-19 patients without vascular thrombosis 2/20 (10%) resulted positive for IgM aCL and anti-β2-GPI antibodies (**Table 3**). None of these 2 samples was confirmed as positive after 12 weeks.

No one of the HD sera studied resulted positive for all the aPLs tested.

Our results indicated that no patient or HD was positive for anti-PF4/polyanion antibodies (**Table 3**).

### Ig fractions isolated from patients with AIS during COVID-19 induce ERK1/2 and p38 phosphorylation

**Figure 1:**
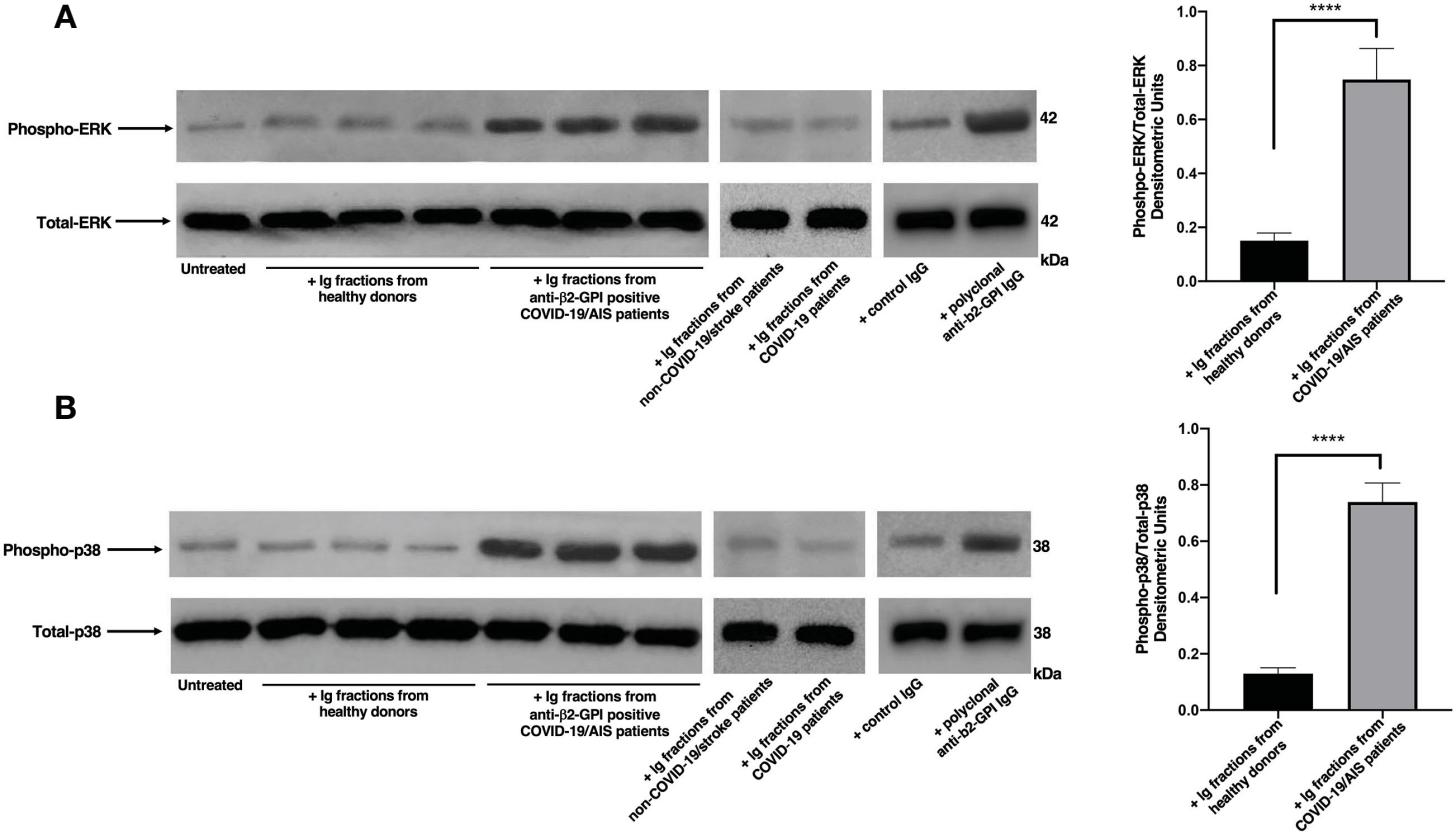
Ig fractions from patients with AIS during COVID-19 induce ERK and p38 phosphorylation. Human platelets from healthy donors were treated for 10 min with Ig fractions (200 μg/ml) from AIS/COVID-19 patients, non-COVID-19 stroke patients, COVID-19 patients without vascular thrombosis and from healthy donors or with polyclonal anti-β2-GPI IgG (200 μg/ml). Protein extracts were separated by SDS-PAGE and analyzed by western blot to investigate phosphorylated ERK **(A)** and p38 MAPK **(B)** using anti-phospho-ERK1/2 and anti-phospho-p38 antibodies, respectively. Samples from three representative healthy donors, three anti-β2-GPI IgG positive patients with AIS during COVID-19 (n. 3, 4, 5 of **Table 2**), one representative non-COVID-19 stroke patient and one representative COVID-19 patient are shown. Densitometric values of phospho-ERK and phospho-p38 levels, calculated in all healthy donors and patients, are represented and summarized by histograms. A statistically significant difference between expression of phospho-ERK and phospho-p38 in patients with AIS during COVID-19 *vs* healthy donors was found (****p < 0.0001).

Since anti-β2-GPI antibodies are the main candidate to trigger platelet activation, platelets from healthy donors were treated with Ig fractions isolated from patients with AIS during COVID-19, with Ig fractions from healthy donors or with polyclonal anti-β2-GPI IgG. As shown in **Figure 1**, treatment of platelets with the Ig fractions from patients with AIS during COVID-19, as well as with polyclonal anti-β2-GPI IgG, induced a significant increase of both phospho-ERK (**Figure 1A**) and phospho-p38 (**Figure 1B**) expression, as compared to control untreated platelets or platelets treated with Ig fractions from healthy donors, non-COVID-19 stroke patients and COVID-19 patients without vascular thrombosis (p < 0.0001). In **Figure S1** the results of all ten patients with AIS during COVID-19 are reported.

### Ig fractions isolated from patients with AIS during COVID-19 trigger ATP release and platelet aggregation

**Figure 2:**
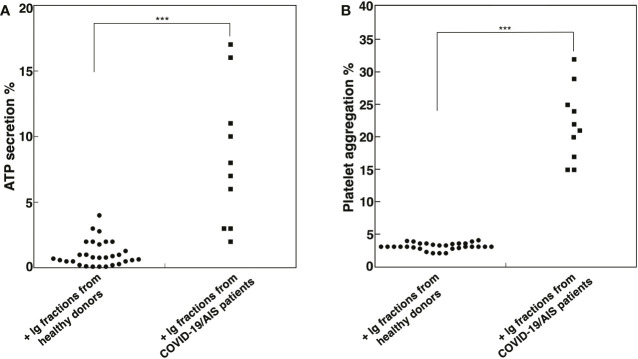
Ig fractions from patients with AIS during COVID-19 induce ATP release and platelet aggregation. Human platelets from healthy donors were treated with Ig fractions (200 μg/ml) from 10 patients with AIS during COVID-19 and from 30 healthy donors. After treatments, platelets were analyzed: **(A)** for ATP release dosage by a luciferin/luciferase method to evaluate platelet secretion; **(B)** for platelet aggregation. Statistical analysis indicates: ***p < 0.001 *vs* healthy donors.

In order to investigate the effect of Ig fractions isolated from patients with AIS during COVID-19 on functional platelet activation, platelets from healthy donors were treated with the Ig fractions. As shown in **Figure 2A**, results highlighted a significant increase of ATP release in samples stimulated with the Ig fractions from patients with AIS during COVID-19 as compared to those from healthy donors (p < 0.001). The analysis revealed that 7 out of the patients with AIS showed a significant increase of ATP release compared to healthy donors, with different values (**Table 2**), summarized in **Figure 2A**. Interestingly, the three patients unable to induce ATP release were resulted negative for all aPL tests. However, the strongest ATP release was with the Ig fraction from the AIS/COVID-19 patient with no aPL Ab, suggesting that antibodies other than aPLs present in sera of COVID-19 patients may activate platelets.

This finding was supported by the analysis of platelet aggregation, which revealed that Ig fractions isolated from AIS/COVID-19 patients induced in platelets from healthy donors an average platelet aggregation of 21.5 + 5.67%, significantly higher of that induced by Ig fractions from healthy donors (p < 0.001), that virtually did not induce platelet aggregation (**Figure 2B**).

## Discussion

This study demonstrates the presence of aPLs in a subgroup of COVID-19 patients who presented acute ischemic stroke during the SARS-CoV-2 infection. Our results converge with the data describing an increased prevalence of aPLs in COVID-19 patients, suggesting a role in the mechanisms contributing to hypercoagulable state observed in patients with critical ill, such as those presenting ischemic stroke (12, 15–17).

Several viral and bacterial infections may lead to both thrombotic and hemorrhagic complications, emphasizing a clear correlation between inflammation and coagulation. Pathogens, as well as inflammatory cells and mediators, are responsible of TF induction, the main initiator of the coagulation cascade (39, 40). In previous works it has been demonstrated an “*in vitro*” pathogenic effect of aPLs, which are able to activate a proinflammatory and procoagulant phenotype in monocytes, endothelial cells and platelets, triggering a signal transduction pathway, leading to proinflammatory cytokines and TF release (41). The severe thrombo-inflammatory manifestations of COVID-19 patients are likely related to pathogenic mechanisms of viral infection and circulating aPLs. Several studies demonstrated platelet activation in COVID-19 patients (42); in particular it was shown that immune-mediated activation of platelets may contribute to the prothrombotic state in these patients (43, 44). With the aim to explore and verify a mechanism directly involved in the amplification of severe COVID-19 disease, we analyzed the effect on a pivotal signaling pattern in human platelets and investigated the functional activation of these cells. Our results showed an activation of MAPK pathways, referred to an increased phosphorylation of ERK1/2 and p38 proteins, following incubation with Ig fractions from sera of patients with AIS during COVID-19, compared to untreated platelets or treated with healthy donor Ig fractions. Moreover, our results highlighted an increase of adenyl nucleotides release, as well as platelet aggregation after treatment with Ig fractions from sera of patients with AIS during COVID-19.

For several years, the homeostatic role of PF4 has been ascertained, since it is released from the alpha granules of activated platelets and it transfers from plasma to the high affinity heparan sulphate on endothelial cells, inhibiting local antithrombin activity (AT), thus promoting clotting (45). In this concern, we additionally tested patient and healthy donor sera for antibodies against PF4/polyanion and no one resulted positive.

It is known that heparin-induced thrombocytopenia (HIT) is caused by antibodies that recognize the PF4 and they have also been found associated with vaccine-induced thrombosis with thrombocytopenia (VITT) syndrome (46). In a recent paper, we analyzed autoantibody specificities in sera from patients affected by VITT, showing the presence of aPF4/polyanion antibodies, as well as aPLs, suggesting a possible pathogenic role (47). Indeed, the aPF4/polyanion antibodies seem to play a role mainly in the thrombotic syndrome occurring after vaccines administration, probably resembling an autoimmune-HIT, where aPF4/polyanion antibodies activate platelets in the absence of heparin. Interestingly, the data of the present study indicate the absence of aPF4/polyanion antibodies in patients with AIS during COVID-19. Thus, in this case, the effect on platelet activation may be triggered by anti-β2-GPI antibodies. However, patients other than anti-β2-GPI positive may activate the signaling pathway. It may be due to the positivity for other antibody specificities (48), including aCL and non-criteria antibodies. In addition, we cannot exclude that antibodies other than aPLs present in sera of COVID-19 patients may activate platelets (49).

In conclusion, our findings indicate that aPLs, including “non-criteria” aPLs, are present in a cohort of patients with AIS during COVID-19, suggesting that these antibodies may represent a thromboembolic risk factor. Moreover, the data on functional and signaling activation in platelets would seek to explain the link between autoimmune response and SARS-CoV-2 viral infection. Further studies will certainly be needed to elucidate the role of aPLs and other immunological mediators in triggering the immunopathological pathways that create the pro-inflammatory and pro-coagulant conditions found in COVID-19. However, detecting these antibodies, as a serological marker to check and monitor COVID-19, may contribute to improve the risk stratification, and drive a personalized treatment with the aim of preventing thromboembolic complications.

## Data availability statement

The raw data supporting the conclusions of this article will be made available by the authors, without undue reservation.

## Ethics statement

The studies involving human participants were reviewed and approved by Local ethical committee (Policlinico Umberto I) number NCT04844632. The patients/participants provided their written informed consent to participate in this study. Written informed consent was obtained from the individual(s) for the publication of any potentially identifiable images or data included in this article.

## Author contributions

FP and MS designed and performed the research. MD and AF selected the patients. GR, SR and VM performed experiments. AL and VM provided and analyzed the data. AC, RM and TG wrote the paper. MS supervised the research and edited the paper. All authors read, edited, participated in the revision, and approved the manuscript. All authors contributed to the article and approved the submitted version.

## Supplementary material

Supplementary Figure 1. Ig fractions from patients with AIS during COVID-19 induce ERK and p38 phosphorylation. Human platelets from healthy donors were treated for 10 min with Ig fractions (200 mg/ml) from 10 AIS/COVID-19 patients. Protein extracts were separated by SDS-PAGE and analyzed by western blot to investigate: (A) phosphorylated and total ERK, using anti-phospho-ERK1/2 and anti-total-ERK1/2 antibodies. (B) phosphorylated and total p38 MAPK using anti-phospho-p38 and anti-total-p38 antibodies.
