# Supplementary figures and images for: Antiphospholipid antibodies in patients with stroke during COVID-19: A role in the signaling pathway leading to platelet activation
Source: Front Immunol. 2023 Mar 2;14:1129201. doi: 10.3389/fimmu.2023.1129201 (PMC10017527; doi:10.3389/fimmu.2023.1129201)

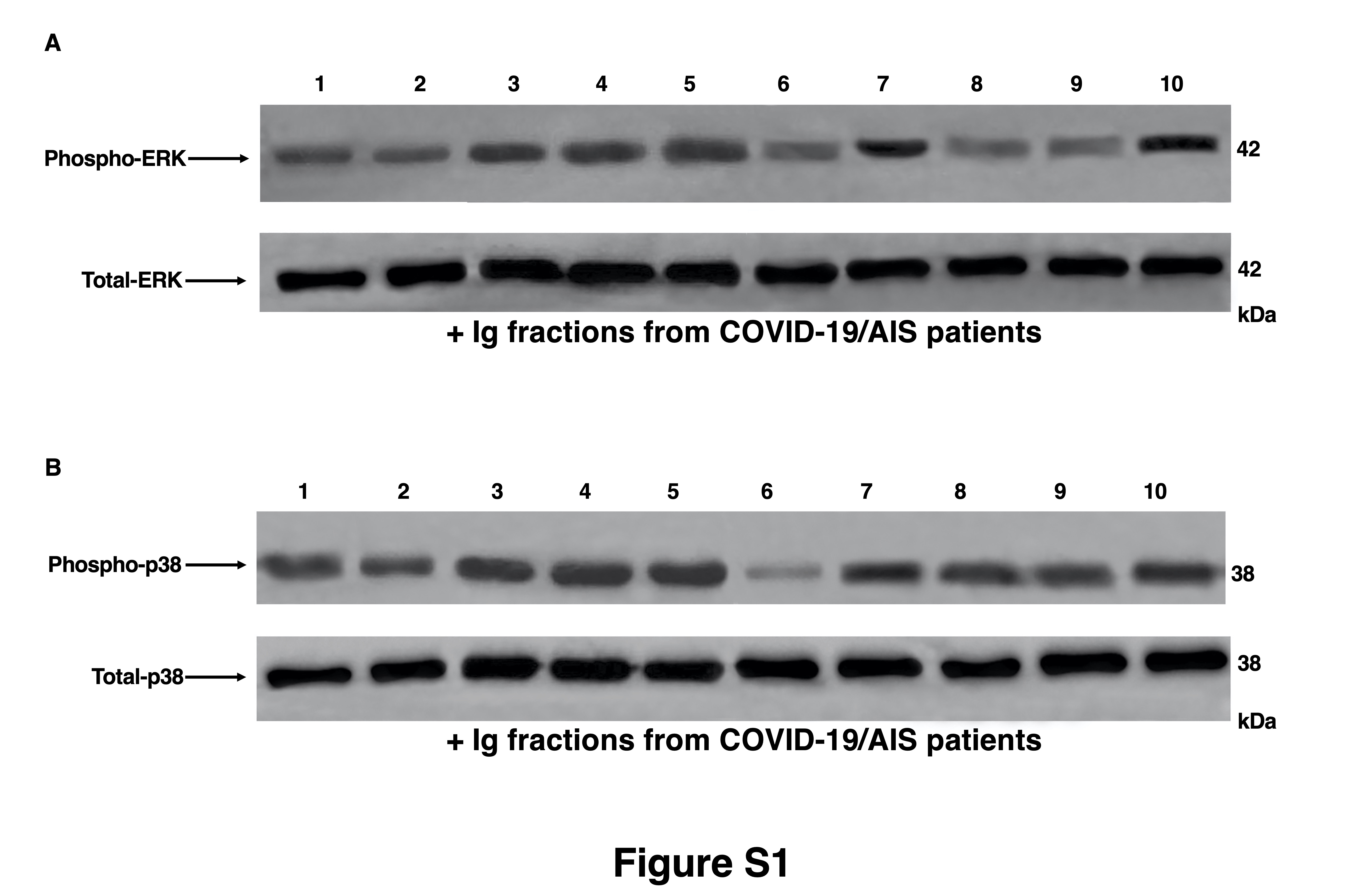

Supplement: Supplementary Figure 1. — Ig fractions from patients with AIS during COVID-19 induce ERK and p38 phosphorylation. Human platelets from healthy donors were treated for 10 min with Ig fractions (200 mg/ml) from 10 AIS/COVID-19 patients. Protein extracts were separated by SDS-PAGE and analyzed by western blot to investigate: (A) phosphorylated and total ERK, using anti-phospho-ERK1/2 and anti-total-ERK1/2 antibodies. (B) phosphorylated and total p38 MAPK using anti-phospho-p38 and anti-total-p38 antibodies. [file Image_1.jpeg]
